# Representativeness of the particulate matter pollution assessed by an official monitoring station of air quality in Santiago, Chile: projection to human health

**DOI:** 10.1007/s10653-022-01390-x

**Published:** 2022-09-20

**Authors:** Margarita Préndez, Patricio Nova, Hugo Romero, Flávio Mendes, Raúl Fuentealba

**Affiliations:** 1 Facultad de Ciencias Químicas y Farmacéuticas, Laboratorio de Química de la Atmósfera y Radioquímica, Sergio Livingstone 1007, Independencia, Universidad de Chile, 8380492 Santiago, Chile; 2 Facultad de Arquitectura y Urbanismo, Laboratorio de Medio Ambiente y Territorio, Universidad de Chile, 8320000 Santiago, Chile; 3 Escuela Superior de Agricultura “Luiz de Queiroz”, Doutorando Em Ciências Florestais, Universidad de Sao Paulo, Piracicaba, Brasil

**Keywords:** Physical characteristics of particulate matter, Spatial heterogeneity, Visible spectrophotometry, Urban geometry, Vegetation cover, Local sources importance

## Abstract

Santiago, capital city of Chile, presents air pollution problems for decades mainly by particulate matter, which significantly affects population health, despite national authority efforts to improve air quality. Different properties of the particulate matter (PM_10_, PM_2.5_ and PM_1_ fractions, particle surface and number) were measured with an optical spectrometer. The sampling was done during spring 2019 at different sites within the official representative area of Independencia monitoring station (ORMS-IS). The results of this study evidence large variations in PM mass concentration at small-scale areas within the ORMS-IS representative zone, which reports the same value for the total area. Results from PM properties such as PM_1_, particle number and particle surface distribution show that these properties should be incorporated in regular monitoring in order to improve the understanding of the effects of these factors on human health. The use of urban-climate canopy-layer models in a portion of the sampled area around the monitoring station demonstrates the influence of street geometry, building densities and vegetation covers on wind velocity and direction. These factors, consequently, have an effect on the potential for air pollutants concentrations. The results of this study evidence the existence of hot spots of PM pollution within the area of representativeness of the ORMS-IS. This result is relevant from the point of view of human health and contributes to improve the effectiveness of emission reduction policies.

**Supplementary Information:**

The online version contains supplementary material available at 10.1007/s10653-022-01390-x.

## Introduction

For decades, Santiago de Chile has been affected by high levels of atmospheric pollution. The outstanding pollutant is the atmospheric aerosol, also called particulate matter (PM), which has relevant effects, on the environment and on human health (Jorquera, 2020). The Health Effects Institute (HEI, 2019) estimates that air pollution (PM_2.5_ and ozone) is the fifth risk factor for mortality worldwide, and in 2017, it was estimated that air pollution contributed to about 5 million deaths worldwide representing almost 10% of total fatalities. Over 55% of the world’s population live in urban areas and this is set to rise to 68% by 2050 (WHO, 2022.

Variability of PM concentrations depends on different factors including local emissions, atmospheric conditions and urban morphology (Buccolieri et al., 2010; Hofman et al., 2013), causing significant spatial and temporal differences in air quality in relatively small areas. Current monitoring network distribution and its expected spatial resolution do not necessarily capture PM variability (Karner et al., 2010; Zikova et al., 2017). Official Representative Monitoring Stations (ORMSs) installed in Chile have a spatial resolution of 2 km radius. However, the official criteria are the population size within these two kilometres, location and sampling conditions (MINSAL, 1998; MMA, 2017). These criteria ignore spatial and temporal pollutants heterogeneity, the distance from the sources and magnitude, the variability of local ventilation and the complexity of urban morphology, contravening the objective of monitoring to determine population daily exposure to pollutants. Inadequate monitoring spatial resolution difficult the estimation of individual exposure to PM, hence offering insufficient information for epidemiological studies of pollutants effects on health; it also does not provide an understanding of the effectiveness of emission reduction policies (Kelly et al., 2017; Tsai et al., 2019), therefore having negative implications for an adequate environmental management.

**Fig. 1:**
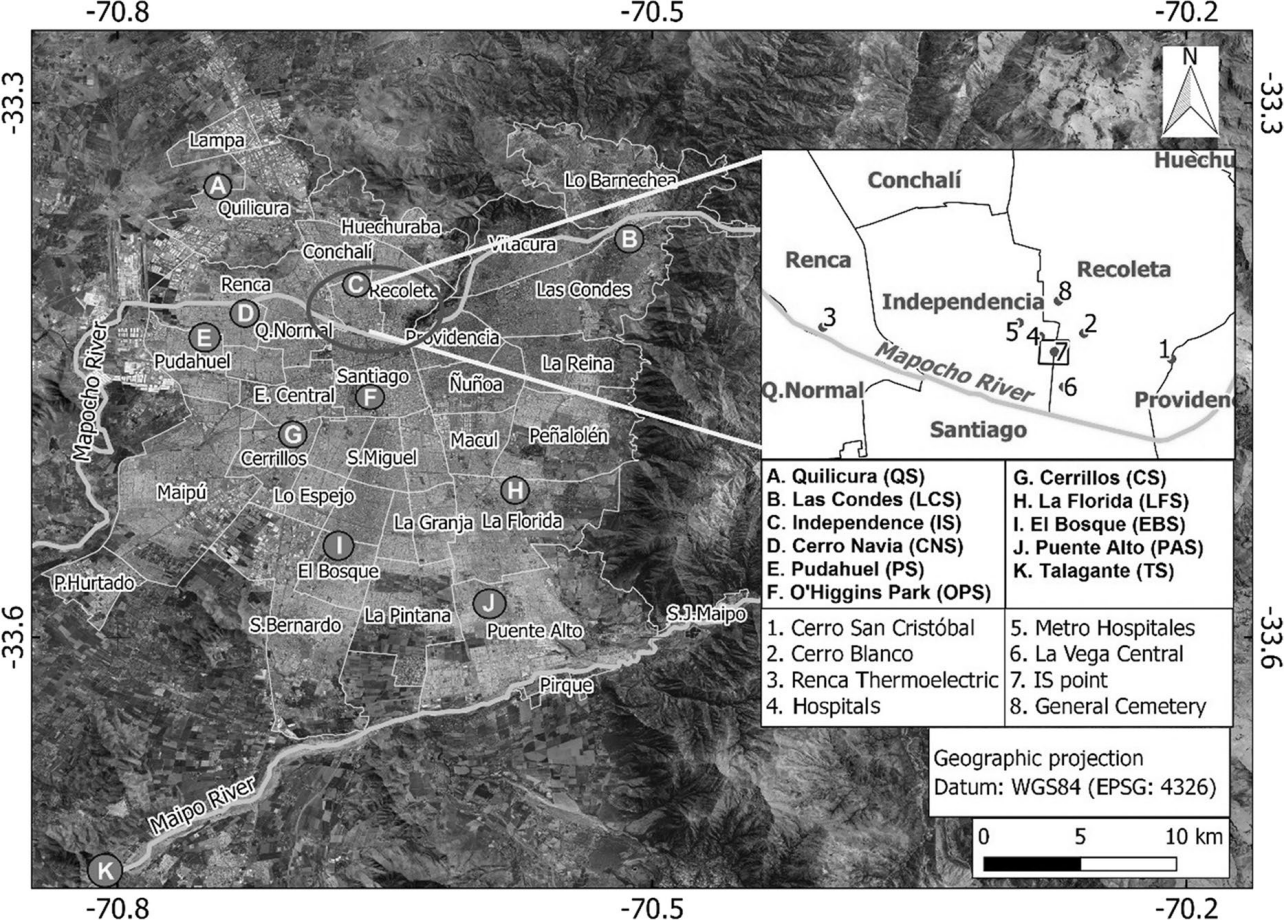
Grand Santiago (grey area), its municipalities (light grey delimitations), and main geographical features around the sampling area (zoomed in box): San Cristobal and Blanco hills, Mapocho River, open market (La Vega) and public services (hospitals, underground subway station, thermoelectric plant and cemeteries) marked with 1–8 numbers, and MACAM-RM network monitoring stations indicated with A–K letters

The National Air Quality Information System (SINCA for its acronym in Spanish) of the Chilean Environmental Ministry (Ministerio del Medio Ambiente, MMA) registers air quality in the country, and it is constantly seeking to improve its monitoring and data management capabilities. In the Metropolitan Region (RM), in which Santiago city is located, the air quality monitoring network known as MACAM-RM has eleven stations (Fig. 1) for continuous particulate matter (PM_2.5_ and PM_10)_, and gases (SO_2_, NOx, CO, O_3_ and non-methane hydrocarbons (HCNM)) measurements.

The MACAM-RM monitoring network uses two types of continuous PM measurement instruments according to national regulations (DS N°59/1998 and DS N°12/2011): Oscillating Conical Element Microbalance (TEOM) and Beta attenuation monitors (BAM). These instruments are expensive with costly operation and maintenance, which limits the possibility of studying the spatial distribution of PM data (Kelly et al., 2017; Wang et al., 2015). The recent availability of instruments based on optical principles of indirect measurement is an interesting alternative to the more expensive instruments used by the MACAM-RM network (Grimm & Eatough, 2009; Holstius et al., 2014; Kelly et al., 2017; Kumar et al., 2015). These alternative instruments are portable and easy to use, which increases the ability to improve PM characterization with high spatio-temporal resolution at a lower cost.

The Metropolitan area of Santiago is divided in 52 municipalities, 34 located in the urban area (or Grand Santiago, henceforth Santiago), and 18 located in rural areas. This study focuses in the urban municipalities (Fig. 1). Besides climatological differences in wind, humidity and temperature (e.g. presence of heat islands) among the municipalities, there is also a marked socio-economical differentiation between the richest areas located to the NE and the rest of the city, where middle- and low-income classes are dominant (Sarricolea et al., 2022).

Hitherto, relevant properties of atmospheric aerosol that have negative impacts on human health, especially in polluted areas, such as highly populated cities, is neither fully investigated nor well understood (Bind et al., 2012; Kuuluvainen et al., 2016; Tsai et al., 2019). Particle number has been associated with adverse health effects such as respiratory diseases among children in urban areas (Li et al., 2016), effects on fibrinogen, i.e. a type of tumour marker (Bind et al., 2012), and in inflammatory markers (Tsai et al., 2019). Systematic studies and meta-analyses (Dinoi et al., 2017; Forlivesi et al., 2018) of the effects of exposure to PM on health have found associations with morbidity and mortality in the population. The above examples complemented with adequate techniques for elemental analysis can be useful to improve physical and chemical characterization of PM (Galvão et al., 2018) and to identify stationary sources that affect a determined area (Fuentealba, 2018; Leoni et al., 2018; Préndez et al., 2007).

Particle surface distribution is emerging as a useful PM property to investigate health effects due to exposure to PM (Kuuluvainen et al., 2016; Ntziachristos et al., 2007). A larger particle surface area could increase the kinetic and thermodynamic potential of chemical reactions in the atmosphere, which can exacerbate pollution. This property is an indication of the internal structure of the particle, such as porosity, that increase particle surface, potentially retaining other pollutants (Cassee et al., 2013; Long et al., 2013; Guo et al., 2017) or even viruses such as SARS-2 (Setti et al., 2020; Zoran et al., 2020), within the particle.

In addition, the monitoring of particle surface distribution contributes to the assessment of pollution reduction policies. Reduction of a 67.2% and 65.0% in particle number concentration and particle surface area has been reported between days with and without vehicle restriction, respectively (Zhao & Yu, 2017). The effect of vehicle circulation restriction on air pollution near a sampling location depends on the distance between the relative orientation of the sampling site and the traffic-restricted areas, as well as on meteorological conditions (Zhao & Yu, 2017).

This study evaluates if an ORMS has the capability to capture i) spatial heterogeneity in PM measurements and ii) urban characteristics of various sampling sites within the station representative area, using a portable visible spectrophotometer to measure PM properties that are not currently quantified by the national monitoring authority.

## Materials and methods

### Study area

The Metropolitan Region of Santiago is located within the basin conformed by Maipo and Mapocho river valleys that descend from the Andes Cordillera, and concentrates about 40% of the country’s population (19 828 563 inhabitants, projection to 30 June 2022, INE, 2022). West of Santiago, the basin is constrained by a coastal mountain range that separates the city from the Pacific Ocean by approximately 130 km. Transversal hills join both mountain ranges substantially reducing atmospheric ventilation at the basin. Since the city is located at a subtropical latitude (33°S), the Mediterranean type of regional climate is under the prevalence of anticyclone influences, and a marked seasonal temperature and rainfall variation, with warm and dry summers and cool and wet winters. Climate at the region, however, has shown a tendency to drier conditions over the last decade due to the ongoing mega-drought that affects central Chile The presence of a persistent radiative thermal inversion layer above the basin explain the remarkable stable atmospheric conditions present over Santiago during the year, which intensify during the winter months. (DMC, 2021). In addition to a large city population, Santiago concentrates most of the country’s industries and services, representing more than 50% of the national Gross Domestic Product (GDP).

The air pollution monitoring station used in this work is located in the Independencia municipality that has around 100 000 inhabitants, positioned in the middle rank of the human development and social indicators (Romero et al., 2010). The monitoring station (ORMS-IS) is part of the MACAM-RM monitoring network. The station is representative of an area that includes parts of Independencia, Recoleta and Santiago municipalities. It is located north of the historical centre of Santiago city and includes many important urban features, for instance, the Mapocho River, the main metropolitan park and zoo (San Cristóbal hill), the largest open market (La Vega) and large cemeteries. Two high traffic roads (Independencia and La Paz avenues) cross the area from north to south and another one (Santa Maria Avenue) from west to east. These avenues connect other municipalities with the city centre and also allow access to several large services such as hospitals, a university campus and an underground subway station. In addition, a thermoelectric plant is located about 1.5 km west of Independencia. During recent years, the predominant middle-class one-story houses have been replaced by tall apartment buildings occupied both by national and migrant population (Fig. 1).

**Fig. 2:**
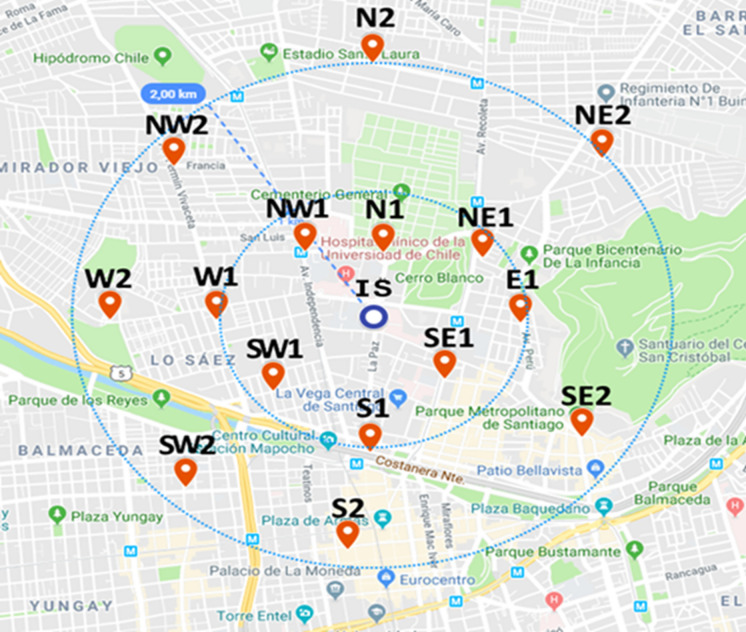
Sampling sites within the representative area of the ORMS-IS

The selected sampling sites in this study locate within a radius of 2 km (Fig. 2) from the ORMS-IS and have the purpose to characterize a population area similar to the area represented by the ORMS-IS.

### Sampling and characteristics of the sampled sites

Sampling was performed during two weeks between 4 and 6 September, and between 10 and 12 September 2019. This month of the year corresponds to the period that follows the critical PM pollution episodes that occur during the austral autumn–winter months (April–August). These episodes relate to various factors that generate low atmospheric PM dispersion (Préndez et al., 2011; Toro et al., 2014). Figure 2 shows the 15 sampling sites reported in this study, labelled according to cardinal direction and distance from the ORMS-IS.

Sampling was performed by a GRIMM spectrometer, Mini-LAS 11-E model with GRIMM Spectrometer 1158-EE Sensor, located 1.75 m above ground level according to the protocol for population exposure to urban pollution (USEPA, 2004). A time resolution of 1 min was used for two periods of 30 min to measure PM_10_, PM_2.5_ and PM_1_ fractions during one period, and the surface distribution and the particle number during the other. The spectrometer has a laser diode with a wavelength in the visible range of 660 nm. The intensity of the laser beam is modulated to detect particles between 0.25 μm and 32 μm and classify them into 31 channels within the range (Grimm & Eatough, 2009).

Vehicle traffic of the study area was obtained using Google Traffic to determine the typical condition or real-time condition in each sampling area. The system detects traffic information, non-moving vehicles, road accidents, and road constructions, using GPS signals of smartphones within each car on the streets. Subsequently, the sampling sites were classified according to vehicle traffic, as shown in Table 1.

**Table 1:** Level of the traffic at each sampling site within the representative area of ORMS-IS. Source: Google Traffic

High traffic	Indicates slow traffic in the sector due to the large number of vehicles circulating at that moment
Medium traffic	Indicates an intermediate level of traffic in the sector
Medium/low traffic	Indicates that sectors with medium and low traffic were observed near the sampling site
Low traffic	Indicates a low level of traffic in the sector and an expeditious travel speed

### Statistics and data

PM_10_, PM_2.5_ and PM_1_ mass concentrations were analysed using Box Plots obtained from the "R" statistical software. Particle number and particle surface distribution were obtained using the Spectrometer_V7-1 Software provided by the Grimm spectrometer and plotted using Excel.

**Fig. 3:**
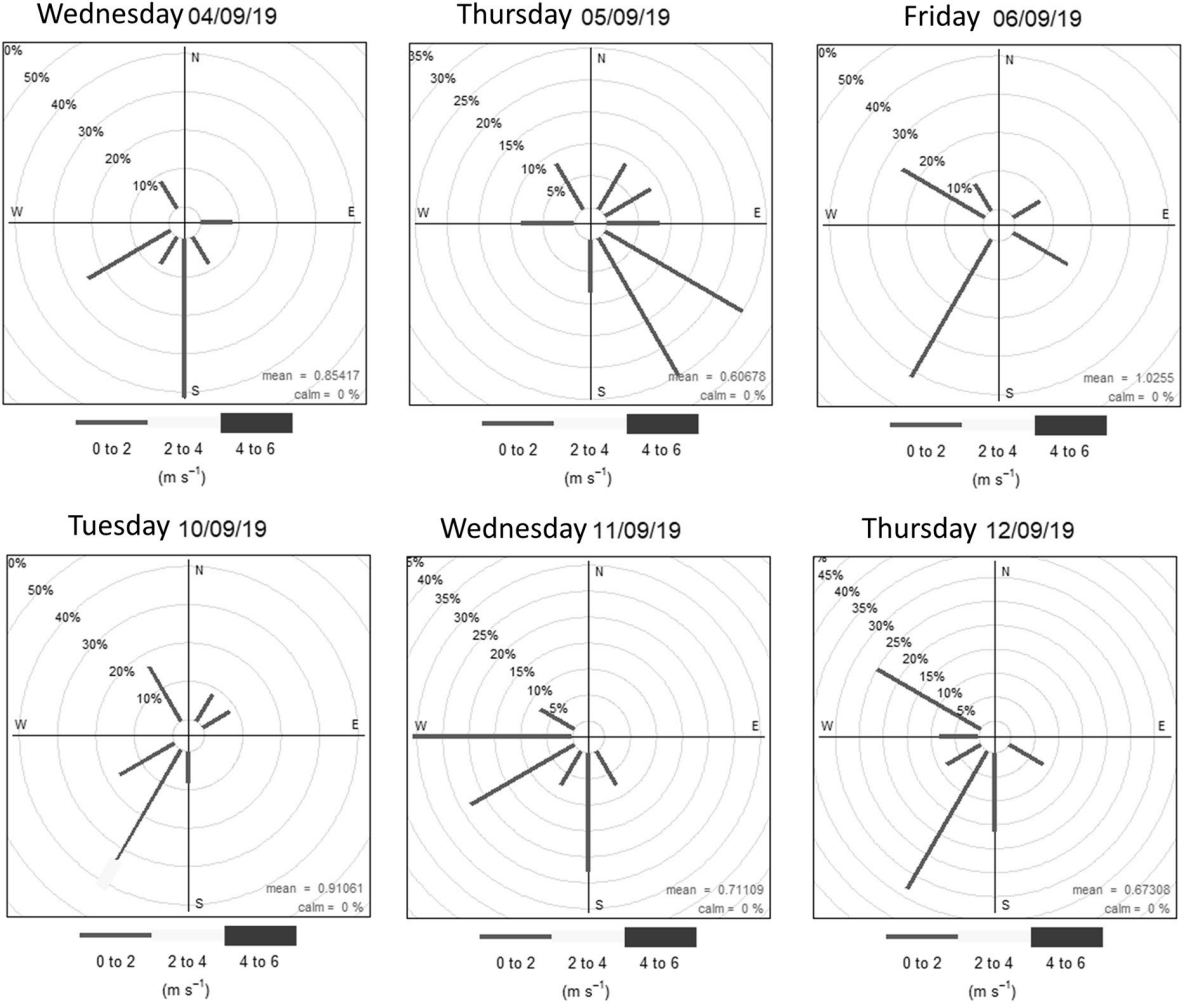
Wind roses according to frequency (%) and wind speed (m s^−1^) during the daytime period 4–6 September and 10–12 September 2019 from 7:00 a.m. to 7:00 p.m. local time

Temperature and relative humidity (RH) were measured using an 1158-EE sensor from the Grimm spectrometer. The temperature effect on the spectrometer’s performance is negligible in the range from 5 °C to 32 °C (Holstius et al., 2014; Zikova et al., 2017). High levels of RH affect the light scattering principle of measurement due to PM absorbing water vapour, therefore changing the dispersion and absorption coefficients (Dinoi et al., 2017). Wind speed and wind direction were obtained from the MMA, (2019) for each sampling day. Figure 3 shows the wind speed and wind direction at 4–5 m height. Wind data were processed using the Open Air extension of the “R” software.

The lognormal function is the most widely used fit to plot PM mass, size and surface distribution because it gives better adjustments for experimental results, being especially useful for the number of particle size ranges greater than 10 units (TSI, 2012). Particle number distribution and particle surface distribution (dN/dlogD) were calculated for the 15 sampling sites during the two sampling periods. *X*-axis and *y*-axis are in logarithmic (base 10) scale (details can be found in the SI.

### Land use and cover, and urban morphology

**Fig. 4:**
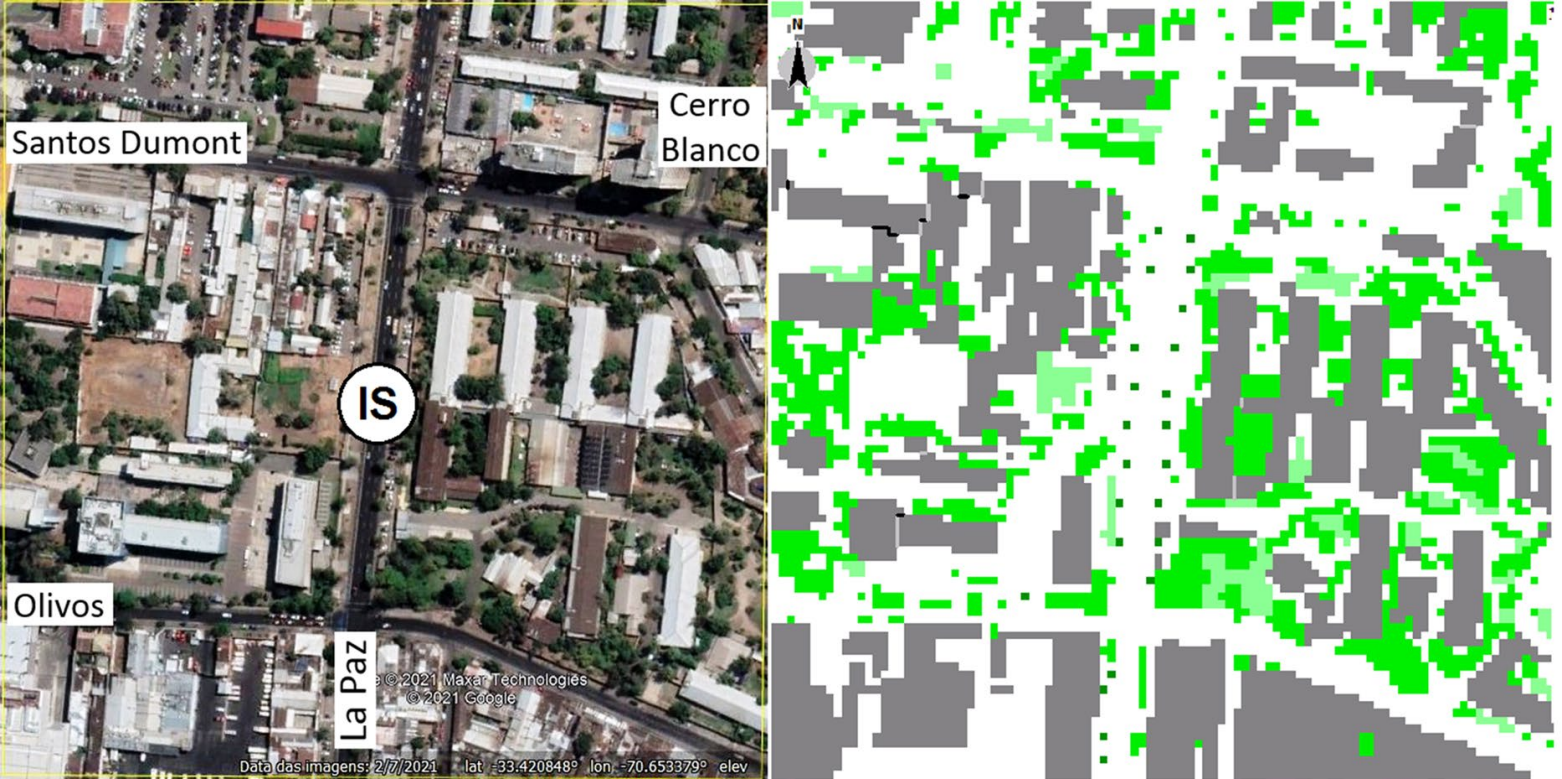
Aerial view and modelled land use and cover at the Independencia municipality (location of the ORMS-IS). Building blocks (grey) cover 33% of the surface, bare land (white) 47.6%, trees (dark green) 15.8%, and grass (light green) 3.3%, respectively

Land use and cover, and the urban morphology around the ORMS-IS were analysed using the ENVI-met model (Mendes et al., 2020). This model considers the spatial distribution of streets, houses and buildings, according to type, position, height, and vegetation cover and density obtained from 2015 Google Street View images. The tallest building in the sampling area reaches 20 floors (~ 60 m height) at Santos Dumont Street near Blanco Hill, but most of the buildings have only one or two floors (between 5 and 6 m height). Vegetation corresponds to palms of 15 m height and lower canopy density (i.e. trees ~ 10 m height), such as *Robinia pseudoacacia*, *Liquidambar styraciflua*, and *Prunus cerasifera*, dense shrubs (~ 6 m height), and grass. Predominant land cover corresponds to sand, followed by asphalt, concrete, and water. The modelled selected area, limited by computational restrictions, corresponds to 400 × 400 m and is located within a 2 km radius from the ORMS-IS (Fig. 2). Figure 4 shows an aerial view of the area (from Google Earth).

## Results

Table 2 shows the main characteristics of the sampling sites.

**Table 2:** Sites sampled description of the local sources and the environment within the representative area of the ORMS-IS between 4 and 12 September 2019

Sites	Traffic	t (°C)	RH (%)	Local sources	Green areas
E1	Mid	22.2 ± 0.1	41.6 ± 0.9	no observation	Cerro San Cristóbal (~ 400 m). Trees on the streets, approximately 3 m near the site
SE1	Mid	22.1 ± 0.6	46.5 ± 0.5	High pedestrian flow (Near a College)	Trees on the streets, approximately 15 m near the site
NE1	Mid/low	15.1 ± 0.4	76.0 ± 1.8	Gas station (~ 30 m). La Recoleta Cinerario (~ 500 m)	Cerro Blanco (~ 70 m) -Cerro San Cristóbal (~ 650 m) Located in a square, approximately 10 m near the site
SW1	Mid/low	15.1 ± 0.3	67.3 ± 1.1	Motor vehicles repair (~ 20 m)	Los Reyes Park (~ 700 m)
NW1	High	14.4 ± 0.3	74.2 ± 0.8	High pedestrian flow (metro entrance). Building (~ 70 m). Cinerario of the General Cemetery (~ 700 m)	no observation
W1	Mid/low	18.9 ± 0.7	59.9 ± 1.5	La Estampa Mill (~ 500 m). Three gas station (~ 150 m / ~ 150 m / ~ 60 m)	Trees on the streets, approximately 10 m near the site
N1	Mid/low	15.8 ± 0.3	68.4 ± 1.0	Building (~ 450 m). Cinerario of the General Cemetery (~ 600 m)	Cerro Blanco (~ 300 m) Trees on the streets, approximately 10 m near the site
S1	Mid/low	15.5 ± 0.4	66.5 ± 2.2	La Vega Market (~ 150 m). Street cooking (~ 100 m)	Río Mapocho Slope (~ 30 m) Trees on the streets, approximately 5 m near the site
SE2	Mid/low	20.4 ± 1.1	54.1 ± 3.0	no observation	Entrance fee of Parque Metropolitano Park (~ 20 m)
SW2	Mid/low	20.6 ± 1.3	50.3 ± 3.5	Gas station (~ 50 m)	Located in a square, approximately 20 m near the site. Los Reyes Park(~ 400 m)
S2	High	15.5 ± 0.4	63.3 ± 1.7	High pedestrian flow (Pedestrian walk)	no observation
N2	Low	26.7 ± 2.1	31.6 ± 0.7	Automotive workshop (~ 50 m)	Trees on the streets, approximately 2 m near the site
W2	Low	16.5 ± 0.7	59.6 ± 1.5	Closest site to Renca thermoelectric power plant (~ 1.7 km). Pharmaceutical industry (~ 700 m)	Enel Stadium (~ 100 m). Los Reyes Park (~ 400 m). Located in a square, approximately 20 m near the site
NE2	Mid/low	26.2 ± 1.2	33.8 ± 0.8	no observation	Trees on the streets, approximately 7 m near the site
NW2	Mid/low	24.6 ± 2.6	42.9 ± 3.4	Pharmaceutical industry (~ 600 m)	Trees on the streets, approximately 5 m near the site

The 15 sampling sites were classified according to typical traffic conditions and sampling time (Table 1). There are two sites with high traffic, two sites with medium traffic, nine sites with medium/low traffic, and two sites with a low level of traffic. It is probable that different levels of traffic at each site contribute to pollutant concentration heterogeneity within the representative area of the ORMS-IS. During the sampling period, temperature ranged from 14.4 ± 0.3 °C to 26.7 ± 2.1 °C, and relative humidity ranged from 31.6% ± 0.7% to 76.0% ± 1.8%. During the sampling period, only one day (10 September) presented a significant amount of rainfall. Wind speed ranged between 0.2 m s^−1^ and 2.3 m s^−1^, with SW, S and W as predominant wind directions.

### PM_10_, PM_2.5_ and PM_1_ mass concentration

**Fig. 5:**
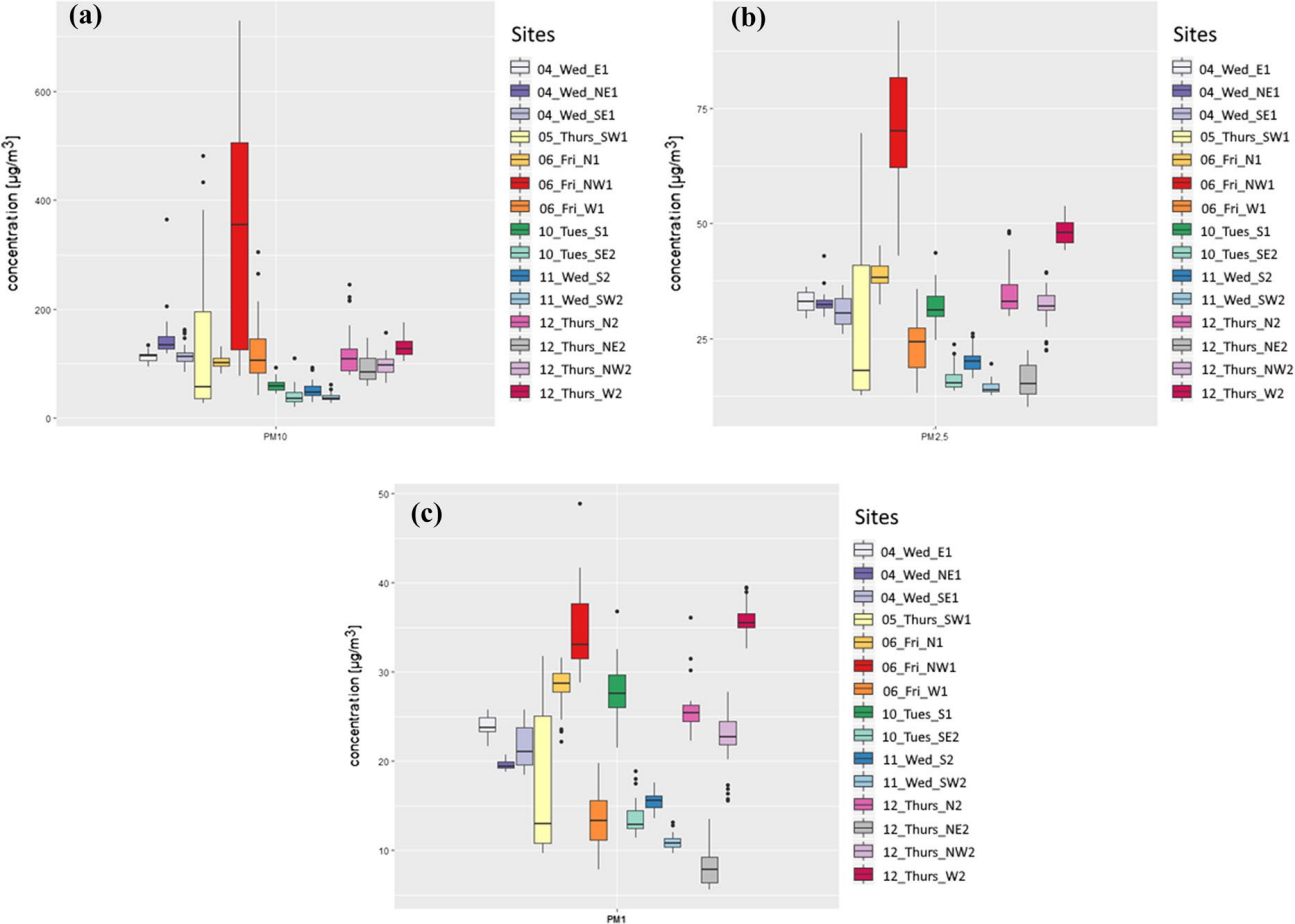
Boxplot diagrams in μg m^−3^ for the 15 sampling sites: **a** PM_10_; **b** PM_2.5_;and **c** PM_1_

Figure 5 (a, b, and c) shows mass concentration of PM_10_, PM_2.5_ and PM_1_. It can be noted that 11 of the 15 sampled sites present outliers. These outliers can be interpreted as high concentrations in time (single data points) due to the high temporal resolution (1 min) of the spectrometric technique employed.

Figure 5a and 5b shows that the highest concentrations of PM_10_ and PM_2.5_ correspond to 6 September at site NW1, with 339 ± 214 μg m^−3^ and 70 ± 13 μg m^−3^, respectively. The minimum concentrations of PM_10_ and PM_2.5_ occurred in 11 September at SW2, with 39 ± 7 μg m^−3^ (almost 9 times lower than the maximum) for PM_10_, and 14 ± 2 μg m^−3^ for PM_2.5_. Site SW2 is located at a vegetated area, which could explain the minimum values obtained on 10 and 11 September. A rainfall event occurred in the early morning of the 10 September.

Additional high concentration of PM_2.5_ occurred at site W2 sampled on 12 September, with (131 ± 16) μg m^−3^ and (48 ± 3) μg m^−3^ for PM_10_ and PM_2.5_, respectively. The W2 site is the closest site from the highway Autopista Central and the Renca thermoelectric power plant (see Fig. 1). On 12 September, NW winds were registered. Three other sites sampled on 4 September show similar levels of PM_2.5_ (29 μg m^−3^ to 33 μg m^−3^). Concentrations of PM_2.5_ on the other sampling days show higher variation depending on location, with a greater spatial variability at the area of representativeness of the ORMS-IS. Figure 5c shows that the W2 site registered the highest concentration of PM_1_, with (36 ± 2) μg m^−3^, followed by NW1, the site with greatest mass concentrations of PM_10_ and PM_2.5_ (Figs. 5a and 5b). PM_1_ and PM_2.5_ have similar sources associated with fossil fuel combustion. The NE2 site, located in a residential sector with no other recorded sources than a medium/low level of vehicular traffic, shows the lowest concentration for PM_1_, with (8 ± 2) μg m^−3^.

### Particle number distribution

**Fig. 6:**
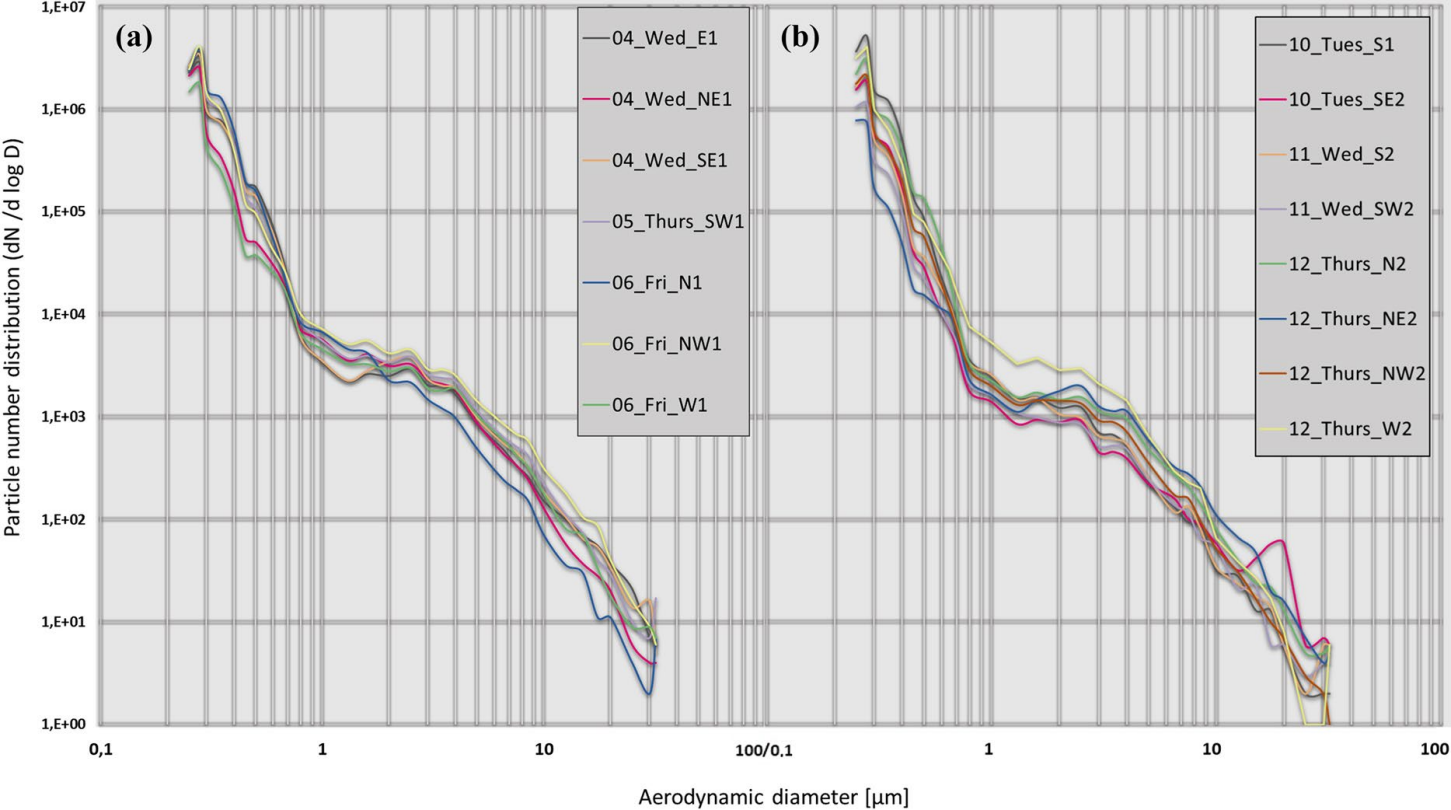
Particle number distribution (dN/dlogD) at the 15 sampling sites: **a** 4 to 6 September 2019; **b** 10 to 12 September 2019. *X*-axis and *y*-axis are in logarithmic (base 10) scale

Figure 6 shows that particle number distribution is mostly below 0.5 μm, i.e. correspond to ultra-fine particles. Figure 6a shows particle number distribution sampled between 4 September and 6 September. The sub-micrometric fraction shows similar distributions for all sampling sites, with NE1 and W1 registering the lowest values, in the range between 0.25 μm and 0.70 μm. Particle number of the PM_1_ fraction fluctuates between 99.5% in W1 to 99.8% in E1 and SE1. The fraction of PM_1-2.5_ shows similar distributions in all sites, with site NW1 being the highest and SE1 the lowest, and in the range between 1.0 μm to 2.0 μm, and the site N1 in the range between 2.0 μm to 2.5 μm. The site N1 located at ~ 700 m from NW1 and sampled the same day shows the lowest particle number concentration, which evidences the spatial variability of concentration in mass and in particle number, in sites located at a short distance. The > PM_10_ fraction shows a greater variability in the distributions than the other fractions.

Figure 6b shows particle number distribution of samples taken on 10 September to 12 September 2019. Particle number percentage registered in the sub-micrometric fraction was between 99.4% (at NE2) and 99.9% (at the sites S1, SE2 and N2) of the total, as in the first sampling week. The sub-micrometric fraction shows that the sites with the highest number of particles are the site S1, in the range of 0.25 μm to 0.45 μm, site N2, in the range of 0.45 μm to 0.60 μm, and site W2, in the range 0.60 μm to 1.00 μm. The < 1.1 μm range (i.e. ultra-fine particles) is linked to fossil fuel combustion such as thermoelectric power plant emissions. As mentioned before, a thermoelectric power plant is located ~ 1.7 km west from the sampling sites. The lowest concentration, between 0.25 μm and 0.60 μm range, was obtained at NE2, without observed local sources and with a medium–low level of traffic. The 0.60 μm to 5.00 μm range shows the lowest concentrations at sites SE2 and SW2, where similar distributions were obtained, which can be attributed to vegetate areas proximity. In the PM_1–2.5_ range, the W2 site shows a particle number higher than other sites. In the PM_2.5–10_ range, W2 shows the highest number of particles between 2.5 μm and 6.5 μm. W2 was exceeded by NE2 in the 6.5 μm to 10 μm range.

### Particle surface distribution

**Fig. 7:**
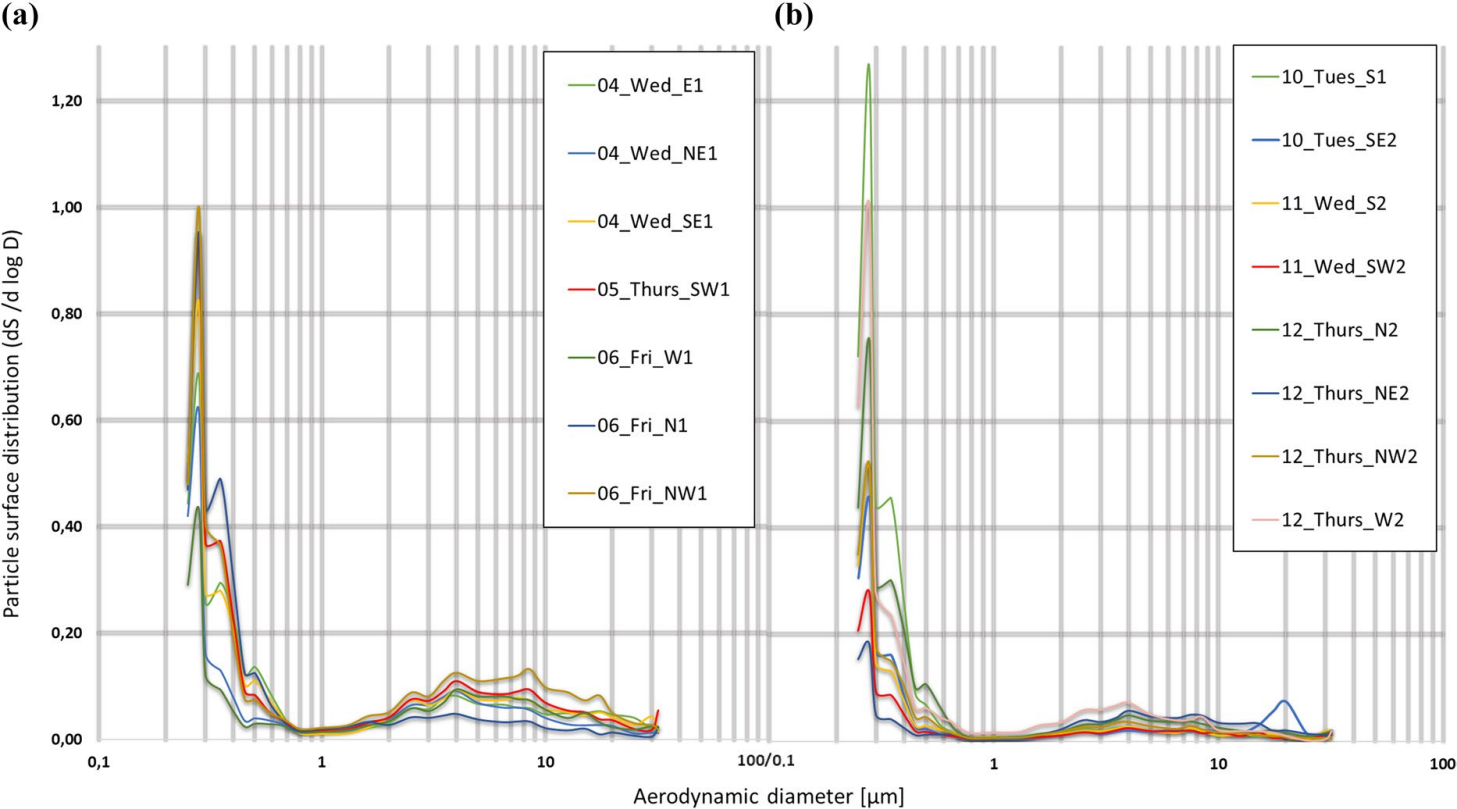
Particle surface distribution (dN/dlogD) at the 15 sampling sites during: **a** 4 September to 6 September 2019; **b** 10 September to 12 September 2019. *X*-axis and *y*-axis are depicted in logarithmic (base 10) scale

Figure 7 shows particle surface distribution of samples taken between 4 September and 12 September. The figure shows two peaks at almost all sites. The first peak is present at all sites, with particles less than 0.3 μm; the second peak is located between 0.28 μm and 0.4 μm. Figure 7a shows the results for the samples taken on 4 September to 6 September. The largest peak is found at all sites at 0.28 μm; a second peak appears at 0.35 μm. There is high distribution variability in the 0.25 μm to 0.65 μm range followed by a convergence of all distribution curves. In the PM_1–2.5_ range, something similar is found. In the PM_2.5–10_ range, the distributions become different again, which may be due to particle number differences (Fig. 6).

**Fig. 8:**
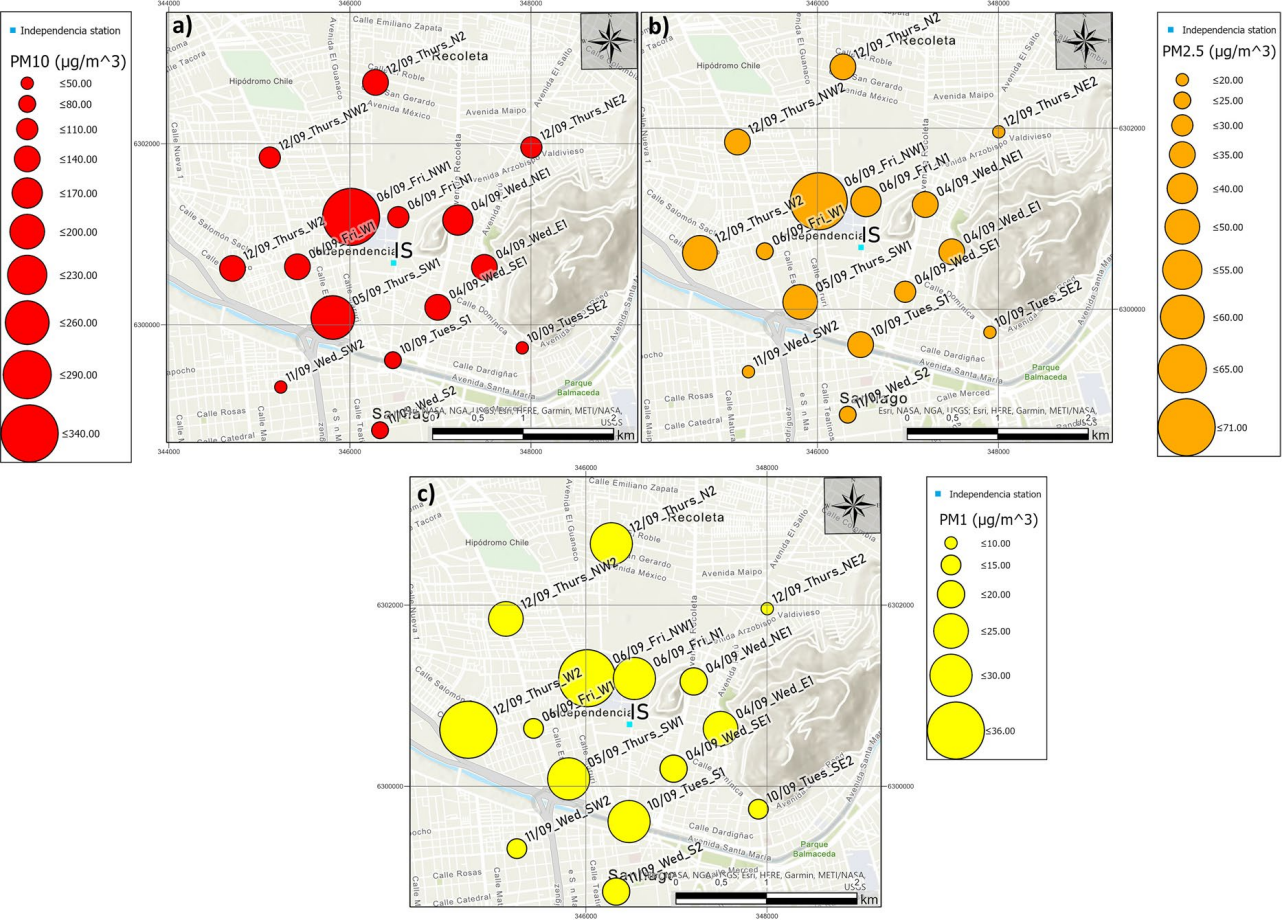
Spatial analysis of the concentrations of PM sampled in the representative area of the ORMS-IS: **a** PM_10_; **b** PM_2.5_ and **c** PM_1_

Figure 7b shows particle surface distributions for the second sampling period. The highest surface percentage is found at the sub-micrometric fraction, accumulating between 55.2% (siteW1) and 85.4% (site N1), of the total PM surface. The largest surface area is concentrated in the sub-micrometric fraction, which accumulates between 47.1% (site NE2) and 92.4% (site S1) of the total PM surface, a more extended range than PM collected during the first sampling period. The surface contribution from the larger aerodynamic diameter ranges is smaller than the observed in Fig. 8a.

The surface maximum is reached at 0.28 μm at all the sites. The observed variability in particle surface distribution is attributable to differences in traffic levels. Particle surface distributions converge in the 0.80–1.30 μm range with minimal surface contribution. Subsequently, the PM_2.5–10_ and PM_10–32_ ranges have a lower surface concentration than the first sampling period. The peak observed at SE2 was also registered in particle number distribution (Fig. 6b).

## Discussion

There is a pharmaceutical industry and a mill located to the NW and W direction from the ORMS-IS (Fuentealba, 2018; Préndez et al., 2007); in addition, the General Cemetery crematorium and La Recoleta Cinerary locate at N and NE from the ORMS-IS, respectively. The above-mentioned industries and services are associated with < 2.5 µm particles emissions (Figs. 1 and 2). The Renca thermoelectric power plant and the industries located in the SW sector of the city (Fuentealba, 2018) can also affect the sampling sites with ultra-fine particles. Previous studies report the presence of Co, Cu, Fe, Na, Sb and Zn in the fine particle size range linked with vehicular emissions (Fuentealba, 2018) in samples collected near the highway Autopista Central. Miler, (2021) in the city of Ljubljana (Slovenia) also found some of those elements coming from vehicle exhaust emissions, brake disc dust and road sediment. Thursday September 5 presented a predominant SE wind direction and lower frequency regimes varying during the day as NW, NE and N (Fig. 3). To note that ultra-fine PM (PM less than 1 μm) can penetrate deeper into the respiratory system (Chen et al., 2016; Shiraiwa et al., 2017), clearly affecting respiratory and cardiovascular systems.

All sampled sites are affected by high, medium or low traffic of personal and public traffic. The highest surface and particle number vales for the site NW1 were found at the PM_2.5–10_ range, which have also the highest PM concentrations.

On the other hand, 11 of the 15 sampling sites are near vegetated areas, including some densely vegetated covers such as San Cristóbal and Blanco hills (located E and NE from the sampling sites) and Los Reyes and Forestal parks (located S and SW from the sampling sites) (Fig. 1). Vegetation contributes to improve air quality due to the capture of PM and the absorption of gases (Escobedo et al., 2011; Gao et al., 2015; Nowak et al., 2013; Préndez et al., 2019). On the > PM10 fraction, a peak was observed at SE2 which coincides with the pollen size range (Ramli et al., 2020). Pollen varies in shape and size (from 10 µm to 100 µm) depending on the species. Two different tree species present at the Metropolitan Park have their pollination period during September–October: *Platanus* x *acerifolia* and *Acer negundo*. The *Cupressus sempervirens* has its pollination period during August–September (PARQUEMET, 2017). In addition to the emission of pollen, site W2 is interesting since it shows the highest number of particles between 2.5 µm and 6.5 μm, and the lowest number of particle surface.

Trees also constitute a natural source of fine PM due to the emission of volatile organic compounds, potential ozone precursors and fine secondary organic compounds (SOA), having a complex chemical composition (Nault et al., 2018; Préndez et al., 2013).

The highest surface percentage of the particle surface distributions is found at the sub-micrometric fraction, which is mostly influenced by anthropic emissions (Gietl et al., 2010; Perrone et al., 2014; Sinha et al., 2011; Zhao & Yu, 2017). However, the percentages are not as high as those observed in the number of particles (99.5% to 99.8%). This is because at the MP_2.5–10_ range, the particles are few in number; however, they represent a significant contribution to particle surface.

Figure 8 shows the spatial analysis of PM concentrations: a) PM_10_; b) PM_2.5_ and c) PM_1_. Figure 8a shows that the highest concentration of PM_10_ and PM_2.5_ corresponds to site NW1 (on 6 September). High concentrations at this site are probably due to the influence of local sources such as high vehicular traffic, re-suspended dust deposited on the pavement, abrasion of vehicle brakes, tyres and pavement degradation, and building construction (Gietl et al., 2010; Thorpe et al., 2007). In addition, the sampling site is in front of Hospitales subway station that presents a large flow of people during daytime. This site also shows the second highest concentration of PM_1_ and a high number of particles of large surface, thus constituting a dangerous site for human health in case of prolonged exposure. However, during the sampling hours, the ORMS-IS station report concentrations of 74 μg m^−3^ and 23 μg m^−3^ for PM_10_ and PM_2.5_, respectively, 4.6 and 3.0 times lower than the observed values in this work ((339 ± 214) μg m^−3^ and (70 ± 13) μg m^−3^, respectively). Concentrations of PM_10_ and PM_2.5_ at site SW1 are lower than NW1, with (230 ± 89) μg m^−3^ and (43 ± 8) μg m^−3^, respectively (Fig. 8b). At the other sites, there are also differences between concentrations of PM_10_ and PM_2.5_. Mean concentrations for 24 h for the daily report by ORMS-IS are 43 μg m^−3^ and 13 μg m^−3^ for PM_10_ and PM_2.5_, respectively (MMA, 2021a, 2021b).

Minimum concentrations of PM_10_, PM_2.5_ and PM_1_ occurred on 11 September at SW2, with (39 ± 7) μg m^−3^, (14 ± 2) μg m^−3^ and (11 ± 1) μg m^−3^. This day registered a mild rain (Figure SI-2), with rainfall often contributing to decrease atmospheric PM concentration (Nowak et al., 2013; Préndez et al, 2014). In the case of ORMS-IS station, SINCA reports concentrations of 23 μg m^−3^ and 3 μg m^−3^ for PM_10_ and PM_2.5_, respectively, during the sampled hours of this work, but does not report accumulative values for 24 h (MMA, 2021a).

PM_1_ concentrations show a completely different spatial distribution than PM_10_ and PM_2.5_. Sites SW1 and W2 show the highest PM concentrations. It is interesting to note that the site W2 increases their mass relative concentration in the sense inverse to the diameter of the particles (Fig. 8c).

Currently in Chile, there are no official standards for the PM sub-micrometric fraction. The lowest PM_1_ concentrations were obtained at the sites SW2, S2, SE2, and NE2, sampled at the ORMS-IS area boundary. The W2 site showed the highest concentration of PM_1_ probably due to its closeness to Autopista Central and Renca thermoelectric power plant, as discussed by Wang et al. (2005) in relation to combustion of fossil fuels.

In the sites located within 1 km from the ORMS-IS, the lowest PM_1_ concentration and particle number was recorded at W1 (at 0.28 µm). This site presents medium/low traffic, without vegetated areas close by, but potentially affected by three gas stations. Using a similar optical spectrophotometer as the one used in this study, Dahari et al., 2021 observed a peak of the number of particles in the submicron range with percentages of the order of more than 95% and attributed these findings mainly to vehicular traffic. Site S1 shows mass concentrations higher than the PM_1_ average (between 17.7% and 58.7%) and the highest number of particles in the range of 0.25 μm to 0.45 μm. This site is the closest to local sources of street cooking that use different combustion types, such as gas stoves for frying or open charcoal grills. The < 1.1 μm range (i.e. ultra-fine particles) is linked to combustion of fossil fuels (Rajput et al., 2016; Wang et al., 2005). In addition, Buonanno et al. (2009) reported the highest percentage of particles from such outdoor cooking coming from activities in the (0.1–1.0) μm range, followed by PM within the (1.0–2.5) μm range. The precariousness of the facilities for street cooking leads to poor combustion; therefore, for this reason, additional research in this topic is necessary to assess the contributing of this PM source.

Independencia municipality, as all the peri-central areas, has increased its urban functionality, density and mobility, during recent years, and it is expected that this trend would continue in the near future. Hospitals, in addition to the largest open market of the city (La Vega), large cemeteries, a large subway station, and heavy traffic roads for private and public transportation have not been the object of systematic environmental planning and management. Vegetation cover is poor and not distributed according to their ecological services. The eventual arrival of clean air masses from nearby hills and parks is constrained by the roughness of an increasing number and density of high-rise buildings. It is necessary for Santiago and other large Latin American cities to incorporate air pollution spatial and temporal detailed information in their sustainable urban development. The presence of such a large number of sources without counterbalancing actions contributes air pollution and increases the population's risk of contracting respiratory and/or cardiovascular diseases.

## Conclusions

New characteristics of aerosol not measured at present by the official air quality monitoring network were quantified within the ORMS-IS area. All 15-sites show the highest values of particle number distributions in the range of ultra-fine particles. Surface distributions at all sites showed that particle size was mostly below 0.4 μm (i.e. nanometric particles), with high potential to absorb other pollutants.

Differences in PM mass concentration, particle number and particle surface distribution were observed within the representative area of the ORMS-IS. PM at the area was most likely due to local sources around the microenvironment, e.g. vehicular traffic, building construction, street cooking and pollen emissions. PM hot spots can be individualized within the studied area, with important implications for human health, including seasonal allergies.

PM_10_ data provided by the ORMS-IS represent well the sites near the station. On the contrary, PM_2.5_ and PM_1_ are not well represented by the ORMS-IS.

The importance of ultra-fine PM (< PM1) and other characteristics of the aerosol such as particle surface and particle number distribution evidence the different quality of air within the monitored area and potential effects on population health. This heterogeneity is the result of local sources, in addition to specific urban conditions and/or the lack of management policies.

The complementary setting of a monitoring network based on instruments assessing the different properties of the aerosol could contribute to improve air pollution policy making at Santiago city.

## Supplementary Information

Below is the link to the electronic supplementary material.

Supplementary file1 (DOCX 130 kb)

## Data Availability

See web page https://figshare.com/articles/dataset/_/16441707
