# Supplementary material for: Representativeness of the particulate matter pollution assessed by an official monitoring station of air quality in Santiago, Chile: projection to human health
Source: Environ Geochem Health. 2022 Sep 20;45(6):2985–3001. doi: 10.1007/s10653-022-01390-x (PMC10232589; doi:10.1007/s10653-022-01390-x)
Supplement: Supplementary file 1 — Supplementary file1 (DOCX 130 kb) [file 10653_2022_1390_MOESM1_ESM.docx]

**Representativeness of the particulate matter pollution assessed by an**

**official monitoring station of air quality in Santiago, Chile: Projection to Human Health**

Margarita Préndez*, Patricio Nova, Hugo Romero, Flávio Mendes and Raúl Fuentealba

**Supplementary Information, SI**

Table SI-1: Date and location when the sampling was performed by the GRIMM spectrometer, Mini-LAS 11-E model.

| Sites | Day | Date | Time | Location |
| --- | --- | --- | --- | --- |
| E1 | Wed. | 04-09-2019 | 13:10-14:23 | Av. Peru & Santos Dumont |
| SE1 | Wed. | 04-09-2019 | 14:40-15:46 | Av. Recoleta & Buenos Aires |
| NE1 | Wed. | 04-09-2019 | 17:32-18:39 | Av. Recoleta &Schlack |
| SW1 | Thurs. | 05-09-2019 | 16:37-17:42 | Escanilla & Aníbal Pinto |
| NW1 | Fri. | 06-09-2019 | 11:44-12:50 | Av. Independencia & Prof. Zañartu |
| W1 | Fri. | 06-09-2019 | 14:51-15:56 | Av. Fermín Vivaceta 838 |
| N1 | Fri. | 06-09-2019 | 13:10-14: 17 | Av. La Paz & Prof. Zañartu |
| S1 | Tues. | 10-09-2019 | 14:25-15: 31 | Av. Santa María & Av. La Paz |
| SE2 | Tues. | 10-09-2019 | 15:53-16: 58 | Pío Nono & Constitución |
| SW2 | Wed. | 11-09-2019 | 15:48-16:58 | Av. Brasil & San Pablo |
| S2 | Wed. | 11-09-2019 | 14:22-15: 28 | Compañia 1140 |
| N2 | Thurs. | 12-09-2019 | 16:08-17: 13 | Av. Einstein & La Conquista |
| W2 | Thurs | 12-09-2019 | 10:42-11: 49 | Gamero & Sara Gajardo |
| NW2 | Thurs. | 12-09-2019 | 12:05-13: 10 | Av. Fermín Vivaceta & Francia |
| NE2 | Thurs. | 12-09-2019 | 17:34-18: 40 | Nicolás de Garnica & Las Torres |

**SI-2: particle size distribution**

The particle size distribution is the size variation of the concentration (in number, surface area, volume or mass) per unit volume of air and can be represented by a differential radius number density distribution, n (r) that represents the number of particles with a radius between r and r + dr per unit volume. See equation 1.

$N\left( r \right)=\int_{r}^{r+dr} n\left( r \right)dr$ Equation 1

In order to study the distribution of sizes, statistical methods are used that simplify their analysis. Using the particle diameter variable D, and on each range or interval of diameters Di - Di + 1 or dD, the number of particles dN is determined according to a histogram, as shown in Figure 3. The data indicates how the number of particles is distributed in a given volume of air (generally 1 m^3^), over the different size ranges considered. This function is called "size distribution" and is noted as dN (D).

The number of channels determine the resolution of a particle spectrometer. The result is dependent on the resolution of the distribution. To prevent distorting the histogram (experimental values) it is normalized by the width of the channel and dividing the number of dN particles in each interval by its width. The area under each channel represents the number of particles, and the height corresponding to each distribution with a different resolution can be compared with the distributions sampled with different instruments. The width of the channel is calculated from the difference of the logarithms at the ends of the decade divided by the number of channels. The normalized concentration is presented on the ordinate axis and the diameter of the particles on the abscissa axis.


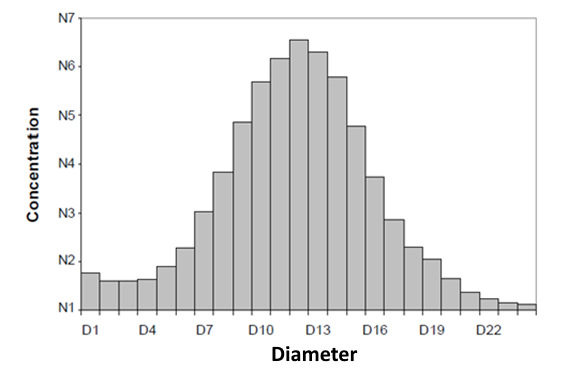


Figure SI-1: Histogram of dN particles concentration in different diameters dD ranges.


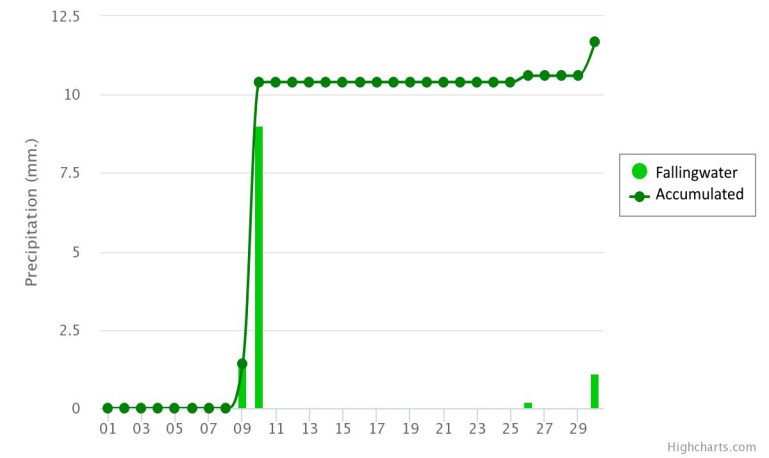


Figure SI-2: Daily (24 hours) and accumulated precipitation, September 2019.

Source: http://archivos.meteochile.gob.cl/portaldmc/meteochile/documentos/Reporte2020_edmay2021_DMC.pdf. Accessed 1 May 2020.

Additionally all dataset of every point analyzed in this article can be find in https://figshare.com/articles/dataset/_/16441707
